# Tumor Treating Fields (TTFields) Hinder Cancer Cell Motility through Regulation of Microtubule and Actin Dynamics

**DOI:** 10.3390/cancers12103016

**Published:** 2020-10-17

**Authors:** Tali Voloshin, Rosa Sara Schneiderman, Alexandra Volodin, Reuben Ruby Shamir, Noa Kaynan, Einav Zeevi, Lilach Koren, Anat Klein-Goldberg, Rom Paz, Moshe Giladi, Zeev Bomzon, Uri Weinberg, Yoram Palti

**Affiliations:** Novocure Ltd., Topaz Building, MATAM Center, Haifa 31905, Israel; tvoloshinsela@novocure.com (T.V.); rosas@novocure.com (R.S.S.); avolodin@novocure.com (A.V.); rshamir@novocure.com (R.R.S.); NKaynan@novocure.com (N.K.); ezeevi@novocure.com (E.Z.); lavigdor@novocure.com (L.K.); akgoldberg@novocure.com (A.K.-G.); RPaz@novocure.com (R.P.); zbomzon@novocure.com (Z.B.); weinberg@novocure.com (U.W.); yoram@novocure.com (Y.P.)

**Keywords:** tumor treating fields, cell motility, rhoa, microtubules, actin, focal adhesions

## Abstract

**Simple Summary:**

Tumor Treating Fields (TTFields), encompassing alternating electric fields within the intermediate frequency range, is an anticancer treatment delivered to the tumor region through transducer arrays placed non-invasively on the skin. Although established as an anti-mitotic treatment modality, the anti-metastatic potential of TTFields and their effect on rapid cytoskeletal dynamics during cellular motility warrant further investigation. In this study, we report that TTFields application induces changes in microtubule organization leading to interference with the directionality and robustness of cancer cell migration. We show that these changes in microtubule organization result in activation of GEF-H1/RhoA/ROCK signaling pathway, and the consequent formation of focal adhesions and changes in actin cytoskeleton architecture. Together, these results propose a novel mechanism by which TTFields induce changes in microtubule and actin organization and dynamics, thereby disrupting processes important for polarity generation and motility in cancer cells.

**Abstract:**

Tumor Treating Fields (TTFields) are noninvasive, alternating electric fields within the intermediate frequency range (100–300 kHz) that are utilized as an antimitotic cancer treatment. TTFields are loco-regionally delivered to the tumor region through 2 pairs of transducer arrays placed on the skin. This novel treatment modality has been FDA-approved for use in patients with glioblastoma and malignant pleural mesothelioma based on clinical trial data demonstrating efficacy and safety; and is currently under investigation in other types of solid tumors. TTFields were shown to induce an anti-mitotic effect by exerting bi-directional forces on highly polar intracellular elements, such as tubulin and septin molecules, eliciting abnormal microtubule polymerization during spindle formation as well as aberrant cleavage furrow formation. Previous studies have demonstrated that TTFields inhibit metastatic properties in cancer cells. However, the consequences of TTFields application on cytoskeleton dynamics remain undetermined. In this study, methods utilized in combination to study the effects of TTFields on cancer cell motility through regulation of microtubule and actin dynamics included confocal microscopy, computational tools, and biochemical analyses. Mechanisms by which TTFields treatment disrupted cellular polarity were (1) interference with microtubule assembly and directionality; (2) altered regulation of Guanine nucleotide exchange factor-H1 (GEF-H1), Ras homolog family member A (RhoA), and Rho-associated coiled-coil kinase (ROCK) activity; and (3) induced formation of radial protrusions of peripheral actin filaments and focal adhesions. Overall, these data identified discrete effects of TTFields that disrupt processes crucial for cancer cell motility.

## 1. Introduction

The high propensity of glioblastoma cells to infiltrate and invade adjacent brain tissue remains as a major obstacle to therapeutic disease management. Therefore, the development of novel treatment modalities that disrupt glioma cancer cell motility could therefore facilitate greater disease control. Tumor Treating Fields (TTFields), encompassing alternating electric fields within the intermediate frequency range (100–300 kHz) [1,2], is a noninvasive, antimitotic cancer treatment delivered locoregionally to the tumor region through 2 pairs of insulated transducer arrays placed on the skin. The intermediate frequency range of TTFields utilized to optimally target various cancel cell types has been previously reported as too high to stimulate nerves and muscles, but low enough so the fields do not significantly heat the tissue [3]. At this frequency, the electric field is able to enter the cell effectively where it exerts a bi-directional force on charged and polar intracellular components, disrupting cancer cell replication [4]. Previous investigations have shown that TTFields disrupt polymerization of highly dynamic microtubules and septin filaments, both of which govern key processes during mitosis [5,6]. Mitotic outcomes elicited by TTFields application include abnormal chromosome segregation, which triggers different forms of cell death [6]. Currently, TTFields therapy is Food and Drug Administration (FDA)-approved for use in patients with newly-diagnosed and recurrent glioblastoma as well as malignant pleural mesothelioma. TTFields is also currently being investigated in other solid tumors in ongoing trials.

Although established as an anti-mitotic treatment modality, the anti-metastatic potential of TTFields and their effect on rapid cytoskeletal dynamics during cellular motility warrant further investigation [7]. Previous studies have demonstrated that TTFields inhibit metastatic properties in cancer cells [8,9,10]. Further elucidating and specifically identifying unified mechanisms connecting the diverse molecular and phenotypic responses observed with TTFields treatment is needed in order to maximally optimize the therapeutic potential of TTFields.

In this study, we report TTFields application induces changes in microtubule organization leading to interference with the directionality and robustness of cancer cell migration, with mechanistic consistency to other anti-microtubule agents and inhibitors of cancer cell invasion [11,12,13,14,15,16]. We show that these changes in microtubule organization result in activation of GEF-H1 and lead to an increase in the levels of active RhoA, activation of Rho-associated coiled-coil kinase (ROCK), and the consequent formation of focal adhesions and changes in actin cytoskeleton architecture. Together, these results propose a novel mechanism by which TTFields induce changes in microtubule and actin organization and dynamics, thereby disrupting processes important for polarity generation and motility in cancer cells.

## 2. Results and Discussion

### 2.1. Migration and Invasion Properties of Cancer Cells are Inhibited by Exposure to TTfields

**Figure 1:**
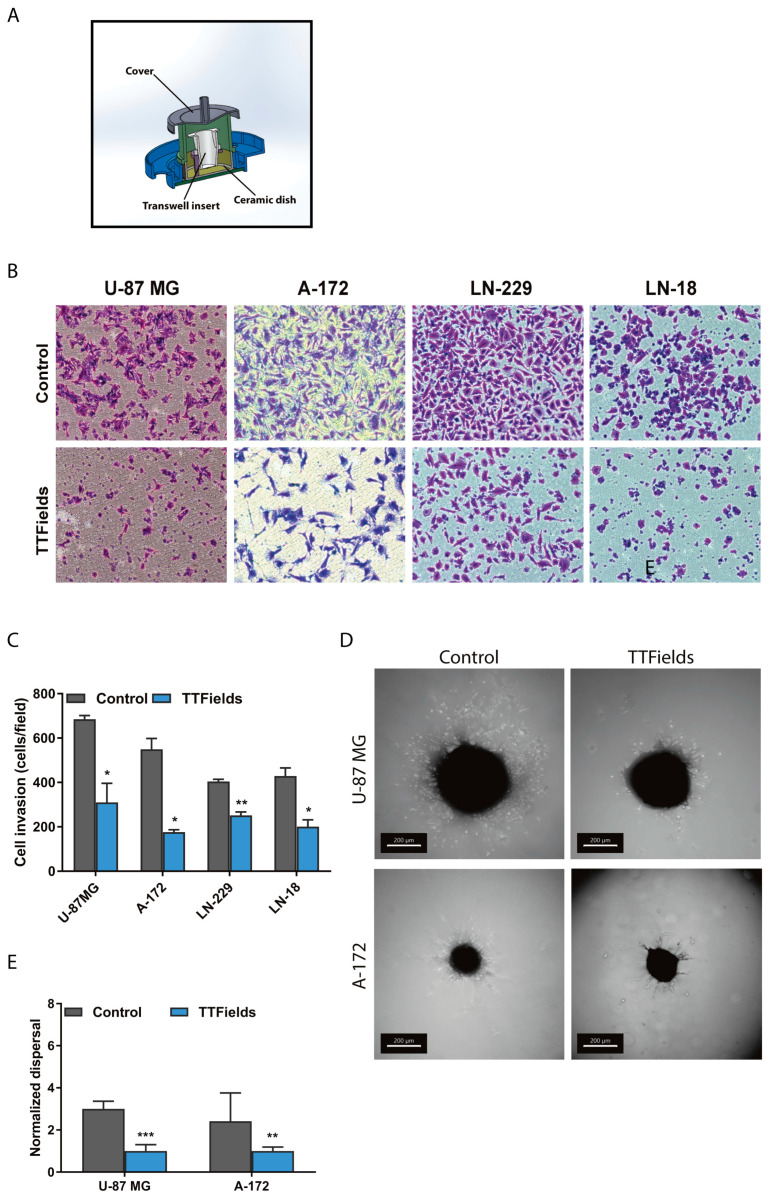
Tumor Treating Fields (TTFields) reduce cancer cell invasion. (**A**) The inovitro^TM^ system with extended wall dish. (**B**) Invasion properties of U-87 MG, A-172, LN-229 and LN-18 glioblastoma cells were evaluated by the modified Boyden chamber assay in the presence of TTFields. Top panel—control cells, bottom panel-TTFields-treated cells (magnification × 100). (**C**) Quantification of invading cells. (**D**) Spheroid dispersal assay of U-87 MG and A-172 spheroids (scale bar: 200 µm). (**E**) Quantification of spheroid dispersal. Mean + SEM; paired *t*-test. * *p*  <  0.05, ** *p*  <  0.01, and *** *p*  <  0.001.

We have previously shown that TTFields application interferes with proper mitotic spindle formation in cancer cells [1,6]. To expand our current knowledge of the effect of TTFields on microtubules and to examine the potential of this local treatment modality as a means to further prevent disease spread, we established a modified Boyden chamber system. This type of system allows for the study and examination of the invasion properties of cancer cells with and without TTFields treatment. For this, TTFields were applied, using the in vitro laboratory research system (Novocure, Israel) with extended wall dishes (Figure 1A).

Human glioblastoma U-87 MG, A-172, LN-229 and LN-18 cell lines were treated with TTFields at a low intensity of 0.6 V/cm RMS and an optimal frequency of 200 kHz for 24 h. Cells that invaded the bottom filter of the chamber were then imaged using phase-contrast microscopy. Noteworthy, the duration of the invasion experiments and the applied electric fields intensities were shorter and lower than the duration and the intensities (above 1 V/cm RMS) required for induction of TTFields anti-mitotic effects [1,2]. TTFields application significantly reduced cancer cell invasion compared to control conditions in all tested cell lines (Figure 1B,C). Subsequently, to evaluate the effects of TTFields on the invasive properties of cancer cells in three-dimensional (3D) models, we embedded spheroids from U-87 MG and A-172 cells in an extracellular matrix blend and assessed their outward dispersal capacity. We observed that TTFields application led to a significant decrease in the dispersal of both U-87 MG and A-172 cells in accordance with the observed effects in the modified Boyden chamber system (Figure 1D,E).

**Figure 2:**
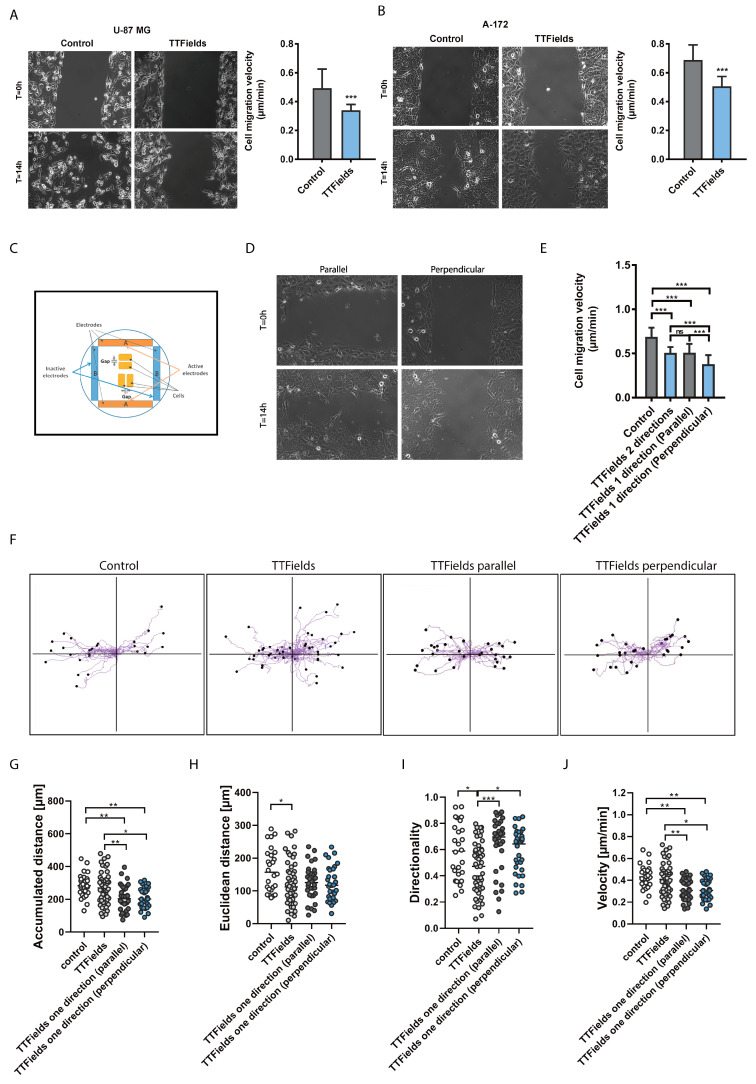
TTFields exposure hinders cancer cell migration (**A**,**B**). A cell-free gap was created in cancer cell monolayers for U-87 MG and A-172 cells (**A**,**B**). Baseline images of the cell-free gap at 0 h and cells that migrated into the cell-free area 14 h later, at the same reference points, are shown for each GBM cell line (A-B left panels). Quantitative analysis of migration rates (velocity) for each GBM cell line are depicted (A-B right panels). Mean + SEM; paired *t*-test. (**C**) Uni-directional application of TTFields cell system set-up is illustrated. (**D**) A cell-free gap was created in A-172 cancer cell monolayer (as in A-B). TTFields were applied uni-directionally using one set of transducer arrays. (**E**) Quantitative analysis of average migration velocity. Mean + SEM; one-way ANOVA, followed by Tukey’s pos*t*-test. ns, not significant. (**F**,**J**) Migratory tracks of single cells exposed to TTFields. (**F**) Right and left tracks indicate, respectively, right and left motion toward the cell-free gap. At least 10 cells from each movie were traced. Bar diagrams for cellular measures of (**G**) accumulated distance, (**H**) Euclidean distance (length of the straight line from start to the endpoint), (**I**) directionality or directional persistence, and (**J**) velocity. Mean; one-way ANOVA, followed by Tukey’s pos*t*-test. * *p*  <  0.05, ** *p*  <  0.01, and *** *p*  <  0.001.

To build on these observations, we examined the impact of TTFields application on cancer cell migration. TTFields are typically delivered through two pairs of transducer arrays that generate perpendicular fields within the treated tumor [7]. To mimic the TTFields settings that are applied in patients, we measured the effect of typical bi-directional TTFields on cell migration rates in wound healing assays. A cell-free gap was created in U-87 MG and A-172 monolayers and cell migration during TTFields application, utilizing the inovitro live system, was monitored using time-lapse microscopy for 24 h. Application of TTFields led to a significant reduction in cell migration velocity compared with untreated control cells (Figure 2A,B; Supplementary Videos S1 [U-87 MG] and S2,S3 [A-172]).

Previous studies have shown that application of an external, direct current electrical field to motile cells can reorient the cells to migrate in the direction of the field in a process known as galvanotaxis [17,18]. To better delineate the directionality elements of TTFields therapy, we applied TTFields uni-directionally relative to the leading edge of the cell monolayer. To ensure that similar TTFields parameters were applied in both directions, A-172 cells were seeded in the same Petri dish, using 2 silicon inserts placed perpendicular to each other (Figure 2C). The inserts were removed after cellular attachment and then uni-directional TTFields were applied using a single pair of electrodes (A-A) with the same TTFields parameters described in Figure 2A,B. Cell migration rate (velocity) was found to be significantly reduced when TTFields were applied perpendicular to the direction of cell movement (toward the gap) as compared to when the field direction of TTFields was applied in parallel to cellular movement (Figure 2D,E). No significant differences were observed in overall migration rates or leading edge velocity, as determined by calculation of the total distance that the cells moved from the edge of the cell-free gap toward the mid-center of the cell-free gap, as a function of time between cells treated with TTFields applied bi-directionally or uni-directionally in parallel with cell movement.

Subsequently, the migratory tracks of A-172 control cells (without TTFields) were compared to those with TTFields treatment in either a bi-directional or a uni-directional application, with the uni-directional application in either a parallel or perpendicular direction relative to the migration path (Figure 2F). No significant differences were observed in the overall accumulated distance between control cells and bi-directional application of TTFields to A-172 cells (Figure 2G). However, under control conditions (without TTFields), untreated individual cancer cells moved along in relatively straight, linear paths as compared with bi-directional TTFields-treated cells. This effect on cell migration patterns was indicated by significant differences in Euclidean distance (Figure 2H) and directionality (Figure 2I), which is defined as a measure of the straightness or linearity of cell trajectories. Significant differences in directionality were also observed between cells treated with bi-directional versus uni-directional TTFields. These observed differences in motility patterns in glioblastoma cells, with or without TTFields treatment, suggest that directional cell motility is hindered by bi-directional TTFields. The velocity of cells treated with uni-directional TTFields was shown to be significantly slower than that of bi-directional TTFields application and control (Figure 2J). This data was in line with the demonstrated reduction in overall accumulated distance (Figure 2G). Consistent with these findings, cells that were treated with TTFields applied perpendicularly to the leading edge of cells showed the lowest average migration velocity (Figure 2E). Interestingly, a significantly higher directionality (directional persistence) was observed in cells treated with uni-directional TTFields (applied in parallel to cell movement) relative to bi-directional application (Figure 2I). To clarify, the decline in velocity elicited by uni-directional application is feasibly compensated for by a significantly more pronounced directionality response in cancer cells. This would suggest that although parallel treatment of cells does elicit slower movement, it is counteracted by a seemingly much stronger directionality response on cell movement. These observations would reasonably explicate the similar average migration velocity of bi-directional versus parallel delivery of TTFields (Figure 2E), likened to the strong directionality effects of the parallel component of bi-directional TTFields delivery.

These results suggest that TTFields exposure hinders cancer cell migration and that a single-direction approach of applying TTFields orthogonal to the path of migration is more effective than parallel application.

### 2.2. TTFields Regulate Microtubule Assembly and Directionality

**Figure 3:**
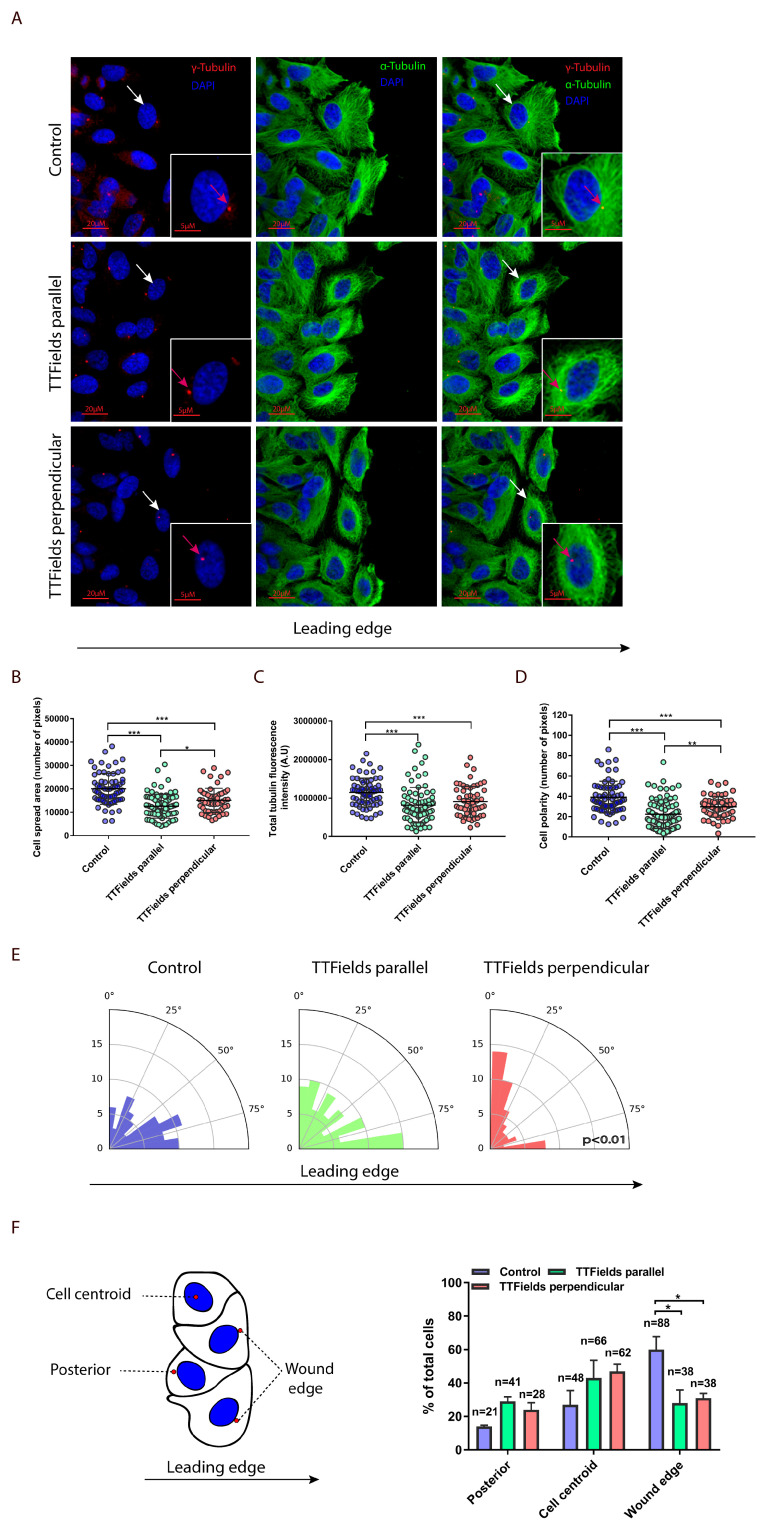
Microtubule assembly and directionality is perturbed by TTFields (**A**) A cell-free gap was created in the LN-299 glioblastoma cell monolayer and TTFields were applied for 7 h uni-directionally, using one pair of transducer arrays (as in Figure 2C–E). Scale bars: 20 µm. Blue, DAPI-stained DNA; Green, α-tubulin; Red, γ-tubulin (microtubule-organizing center (MTOC)). White arrows indicate cells in small micrographs, Magenta arrows indicate MTOC. Quantitative analysis are depicted of: (**B**) cell spread area, (**C**) total sum of tubulin fluorescence within the cell, (**D**) cell polarity that is defined as the distance from cell nucleus to center of mass, and (**E**) average cell angle relative to the leading edge (90°). Y-axis represents the number of cells. Mean + SD; one-way ANOVA, followed by Tukey’s pos*t*-test. (**F**) Diagram of the wound healing assay showing (left panel) the position of the MTOC (red) relative to the nucleus (blue) and (right panel) the frequency distribution of the position of the MTOC relative to the nucleus and the leading edge. Mean + SEM; two-way ANOVA, followed by Tukey’s pos*t*-test. * *p*  <  0.05, ** *p*  <  0.01, and *** *p*  <  0.001.

Based on the aforementioned finding, additional questions arose. How does TTFields directionality affect cellular motility? And more broadly, does the effect of TTFields on microtubules also elicit other cytoskeletal changes? To address these questions, the organization of the microtubules at the leading edge of the wound were examined. Using the inovitro live system, TTFields were applied uni-directionally to LN-299 cell monolayers. Treatment duration was reduced to 7 h to limit the spatial locomotion of cells, following release from contact inhibition and to enforce directional progression (Figure 3A).

Microtubule staining and confocal fluorescence microscopy of cells at the wound edge revealed that application of TTFields in parallel to the direction of the leading edge induced perinuclear localization of the microtubules (Figure 3B). In both directions, the intensity of fluorescence collected from the labelled microtubules was significantly lower than what was observed in the control group (Figure 3C). This confirms the general reduction in the quantity of microtubules that was previously described, using biochemical analysis of the ratios of polymerized to total intracellular tubulin in TTFields-treated cells [6]. Application of TTFields in parallel to the direction of the leading edge also led to a reduction in cell polarity (defined as the distance from cell nucleus to the center of mass; Figure 3D). Moreover, TTFields applied perpendicularly to the leading edge resulted in reduction in the cell spread area (Figure 3B) and cellular polarity (Figure 3D), in addition to eliciting alignment of the microtubules in the direction of the electric field (Figure 3E). Collectively, these data support the findings that TTFields hinder the assembly of microtubules and dictate their directional assembly during cell movement. Interestingly, the position of the microtubule-organizing center (MTOC) was also affected by TTFields. During cell polarization, previous scratch-wound studies showed that the MTOC remains centered, while the nucleus relocates to a more posterior position relative to the leading edge of the cell. Hence, in a typical polarized morphology, the MTOC is positioned between the nucleus and the leading edge [19,20]. Accordingly, in cells evaluated under control conditions, we found that the MTOC was primarily positioned between the leading edge and the nucleus (wound edge; Figure 3F). However, the frequency of cells with this MTOC reorientation were significantly lower in TTFields-treated cells, with posterior (MTOC localized between the nucleus and the rear of the cell) and centroid orientations being more prominent. Since cellular polarization is mainly determined by the location of the MTOC relative to the nucleus, these results suggest that cellular polarization is altered under TTFields application.

### 2.3. Microtubule Disruption in TTFields-Treated Cells Re-Organizes the Actin Cytoskeleton and Induces Formation of Focal Adhesions

**Figure 4:**
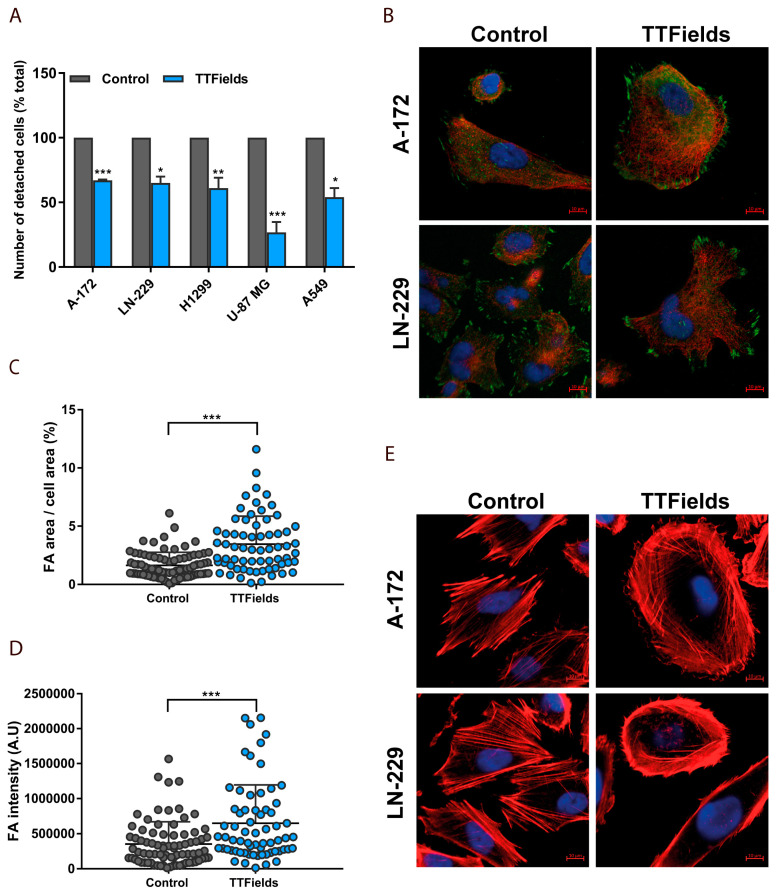
TTFields induce formation of focal adhesions (FA) and reorganization of the actin cytoskeleton in glioblastoma and lung adenocarcinoma cells (**A**) U-87 MG, A-172, and LN-229 glioblastoma cell lines and A549 and H1299 lung adenocarcinoma cell lines were treated continuously with TTFields for 24 h. Kinetics of cell surface detachment demonstrated a difference in surface binding without TTFields (control) and with TTFields treatment. The bars indicate selected time point for each cell line. Mean + SEM; paired *t*-test. (**B**) A-172 and LN-229 cells were treated with TTFields for 24 h. Confocal fluorescence microscopy images of vinculin, a common marker of focal adhesions in control cells (left panels) and TTFields-treated cells (right panels) are shown. Blue, DAPI-stained DNA; Red, Tubulin; Green, vinculin. Scale bars: 10 µm. Assessment of focal adhesion parameters (**C**) Quantification of focal adhesions area relative to cell area in LN-229 cells. Mean + SD; paired *t*-test. (**D**) Quantification of focal adhesions intensity. (**E**) A-172 and LN-229 cells were fixed and stained for F-actin following application of TTFields for 24 h. Left panel, control cells. Right panel, TTFields-treated cells. Blue, DAPI-stained DNA; Red, Phalloidin-stained Actin. Scale bars: 10 µM. * *p*  <  0.05, ** *p*  <  0.01, and *** *p*  <  0.001.

To further dissect the mechanism by which TTFields interfere with cancer cell motility, changes in cell-surface interactions under TTFields application were examined. Multiple cancer cell lines were treated with TTFields for 24 h, then a cellular detachment assay was performed. The assay assessed the number of removed cells after varying trypsinization times, by calculating the fraction of detached cells over the total number of cells in the dish. TTFields reduced cellular detachment from polystyrene in all TTFields-treated cancer cell lines compared with untreated control cells (Figure 4A).

These results suggest that TTFields exposure potentiates cellular adherence of cancer cells. To demonstrate this effect explicitly, we used confocal fluorescence microscopy to examine possible changes in focal adhesions. A-172 and LN-229 cells were TTFields-treated for 24 h and then stained for the focal adhesion protein vinculin. Subsequently, quantitative parameters of focal adhesion patterns using image analysis were assessed. TTFields application was found to result in a dramatic increase in focal adhesion size and numbers as well as peripheral distribution of the adhesion sites (Figure 4B–D). Treated cancer cells adopted a more spread shape in comparison with control cells. Interestingly, F-actin staining revealed that while control cells were highly polarized, TTFields-treated cells exhibited a dense meshwork of actin filaments around the entire cell periphery, resembling the appearance of dorsal stress fibers and transverse actin arcs (Figure 4E). These results suggest that TTFields application results in radially oriented adhesions and complete re-organization of the actin cytoskeleton of treated cancer cells.

### 2.4. TTFields-Induced Microtubule Disruption in Glioblastoma Cells Regulates GEF-H1 to Activate RhoA Signaling

**Figure 5:**
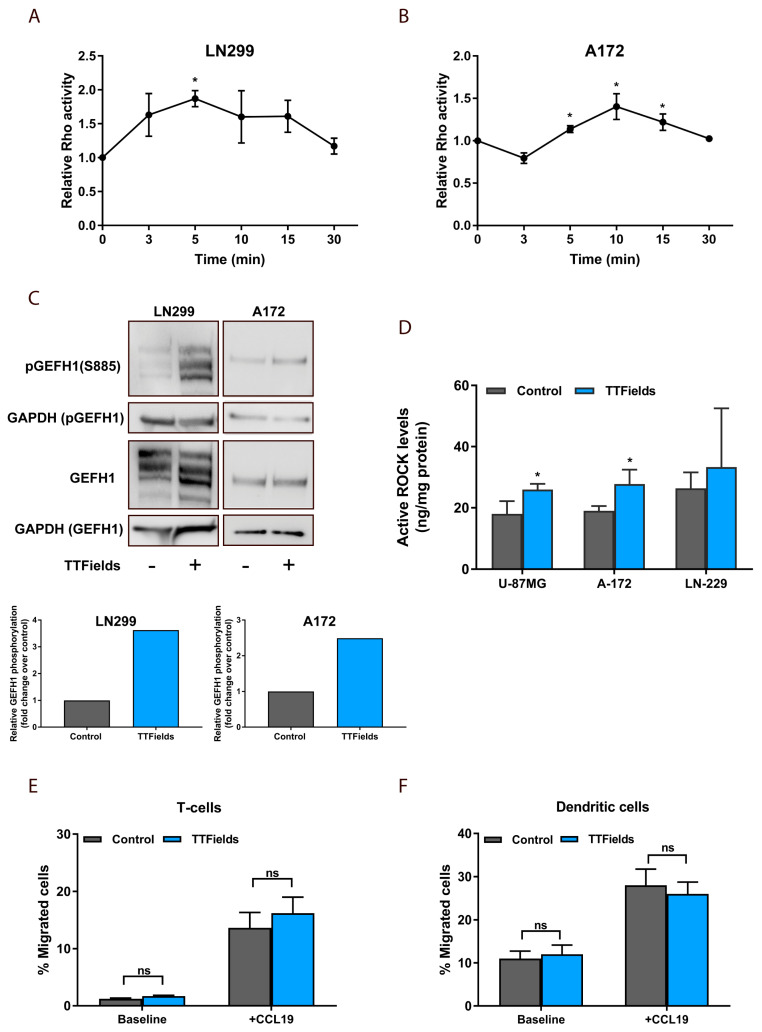
TTFields activate GEF-H1 and inhibit cancer cell motility in a Rho-dependent manner (**A**,**B**) A-172 and LN-229 glioblastoma cells were treated with TTFields for the indicated times and RhoA activation was assessed. Mean + SEM; paired *t*-test. (**C**) Western blot analysis of GEF-H1 phosphorylated on Ser886 and the total GEF-H1 levels in control and TTFields-treated cells (top panel). Quantification of normalized relative levels of P-GEF-H1 compared to total GEF-H1 levels from two western blots for each condition (bottom panel). (**D**) ROCK activity in A-172, LN-229, and U-87 MG cells treated with TTFields for 24 h. (**E**,**F**) Migration responses of (**E**) T-cells and (**F**) dendritic cells using the modified Boyden chamber either with or without CCL19. ns, not significant. Mean + SEM; paired *t*-test. * *p*  <  0.05.

Given the morphologically distinct phenotype of cancer cells following TTFields application, signaling pathways that regulate actin dynamics and focal adhesion formation were subsequently examined. The small GTPase RhoA plays a central role in the regulation of stress fiber assembly and the induced formation of focal adhesions [21,22]. Hence, the effects of TTFields application on microtubule disruption, perhaps promoting the activation of RhoA to control changes in focal adhesions and the re-organization of the actin cytoskeleton, was evaluated [13,23,24]. RhoA activation was assessed in A-172 and LN-229 cells following TTFields application for 3, 5, 10, 15, and 30 min, using the RhoA G-LISA activation assay. Results demonstrated that TTFields application induced a significant and transient increase in RhoA activity (Figure 5A,B).

To determine if TTFields-induced changes in RhoA activity are mediated by microtubule-associated GEF-H1, we evaluated changes in the phosphorylation of the GEF-H1 on Ser886 in A-172 and LN-229 cells exposed to TTFields via Western blot analysis [25,26]. Albeit multiple guanine nucleotide exchange factors have been shown to activate Rho GTPases, GEF-H1 was of focus since its catalytic activity toward RhoA is downregulated through microtubule binding [24,27]. Consistent with the above hypothesis, TTFields application was demonstrated to promote phosphorylation of GEF-H1 (Figure 5C). Subsequently, activation of ROCK, which mediates the downstream effects of RhoA on stress fibers and focal adhesions, was evaluated and showed that TTFields exposure significantly increased the activity levels of ROCK in treated cells (Figure 5D) [23]. Jointly, these data show that the RhoA/ROCK signaling pathway is an essential component of signal transduction pathways linking TTFields-induced microtubule disruption to the induction of peripheral actin bundling and focal adhesion formation.

RhoA has been previously reported to serve as a key regulator of leukocyte differentiation and function [28]. Recently, we demonstrated that TTFields induced immunogenic cell death in cancer cells and initiated an adaptive immune response in vivo [29]. Therefore, to assess whether TTFields–dependent activation of RhoA also has a direct effect on leukocyte migratory function, we compared chemotactic responses of isolated leukocytes in vitro. We examined migration responses of bone marrow derived dendritic cells (BMDCs) and splenic T-cells using the modified Boyden chamber either with or without CCL19 (chemoattractant), which promotes leukocyte recruitment and migration [30,31]. When TTFields were applied at the optimal frequency of 200 kHz, no differences were observed relative to control conditions in the number of leukocytes that relocated in random migration (without CCL19) and due to CCL19-induced migration (Figure 5E,F). These results suggest that TTFields application at 200 kHz did not impair random or chemoattractant-induced leukocyte migration.

## 3. Materials and Methods

### 3.1. Lung Adenocarcinoma and Glioblastoma Tumor Cell Lines

All cell lines were obtained from the American Tissue Culture Collection (ATCC, Manassas, VA, USA). Human lung adenocarcinoma cell lines H1299 and A549 were grown in RPMI Medium. Human glioblastoma cell line U-87 MG, was grown in Eagle’s Minimum Essential Medium. Human glioblastoma cell lines A-172, LN-229, and LN-18 were grown in Dulbecco’s modified Eagle’s medium. All culture media were supplemented with 10% or 5% (LN-229) (*v*/*v*) fetal bovine serum, 2 mM L-glutamine, and penicillin/streptomycin (50 μg/mL). All cells were incubated in a humidified incubator supplied with 5% CO_2_. Standard cell phenotypes were checked and did not change over time.

### 3.2. TTFields Application In Vitro

TTFields were applied using the in vitro™ system (Novocure, Haifa, Israel) as previously described [32]. Cells were treated with TTFields at an intensity of 1.75 V/cm (RMS) and at optimal frequencies of 150 kHz for lung adenocarcinoma cell lines (H1299 and A549) and 200 kHz for glioblastoma cell lines (U-87 MG, A-172, LN-229, and LN-18).

### 3.3. Cancer Cell Invasion Assay

Invasion assay was performed using modified Boyden chamber. TTFields were applied using the inovitro system with extended wall dishes. Filters (6.4 mm in diameter, 8 μm pore size) coated with Matrigel (Corning, Glendale, AZ, USA) were used. Cells (2 × 10^5^) were suspended in serum-free DMEM and seeded in the upper chamber compartment. The lower compartment contained 10% FBS DMEM. After incubation for 24 h at 37 °C, in a 5% CO_2_ incubator, cells that invaded into the bottom chamber filter were fixed with 4% Paraformaldehyde (PFA) and were stained with 0.5% crystal violet (Sigma-Aldrich, Rehovot, Israel). Cells were imaged utilizing an inverted microscope (Nikon eclipse TS100, objective × 10). Quantification of invading cells was performed using FIJI ImageJ software (NIH, Bethesda, MD, USA).

### 3.4. Wound Healing Assay

In vitro wound healing assays were performed, using ibidi culture inserts (ibidi GmbH, Gräfelfing, Germany). TTFields (200 kHz, 1.75 V/cm RMS) were applied using the inovitro live^TM^ system. In the experiments in which TTFields were applied bi-directionally, TTFields changed direction by 90 degrees every 1 s. Migration was observed in time-lapse series for 14 h (Zeiss axio observer; per 10 objective; Carl Zeiss Microscopy GmbH, Jena, Germany). Phase contrast images were recorded every 12 min. The obtained images were further evaluated to quantify cell migration rates during wound healing using the Image Pro Premier (Media Cybernetics, Rockville, MD, USA) software. Quantitative analysis of the migration rate was calculated as the total distance that the cells moved, from the edge of the cell-free gap toward the mid-center of the cell-free gap, as a function of time. Migration tracks of individual cells were obtained using the Manual Tracking plugin of the Fiji software. Migration parameters (accumulated distance, Euclidean distance, directionality, and velocity) were obtained with the Chemotaxis and Migration tool from ibidi.

### 3.5. Immunofluorescence

For microtubule staining, LN-299 (glioblastoma) cells were grown on glass cover slips and treated for 7 h, using the inovitro system. At treatment cessation, cells were fixed with 4% paraformaldehyde (PFA) for 15 min. Subsequently, cells were washed 3 times with PBS, blocked with donkey-serum blocking solution for 1 h, and then stained with anti-tubulin antibody (rabbit polyclonal, Abcam ab18251, Cambridge, UK). Alternatively, at treatment cessation, cells were fixed with ice-cold methanol at −20 °C for 7 min. Then, the cells were washed 3 times with TBST and blocked with blocking solution containing 5% BSA in TBST for 1 h, and stained with gamma tubulin (rabbit polyclonal, Abcam ab11317) and alpha tubulin (mouse monoclonal, Sigma-Aldrich T5168).

For staining of vinculin and actin, A-172 and LN-229 (glioblastoma) cells were grown on glass cover slips treated with bovine fibronectin (1:100, Biological Industries, Beit HaEmek, Israel) and using the inovitro system treated for 24 h with TTFields. Cells were then serum-blocked and stained with mouse anti-vinculin (1:200, mouse monoclonal; Abcam). Donkey anti-mouse Cy3 (1:400), Donkey anti Rabbit IgG Alexa fluor 647 (1:400), Donkey anti-Rabbit IgG Alexa fluor 647 (1:400) and Donkey anti-Rabbit IgG Alexa fluor 488 (1:400) secondary antibodies (Jackson ImmunoResearch, West Grove, PA, USA) as well as Phalloidin (Rhodamine, 1:1000; Cytoskeleton, Denver, CO, USA) were used. DNA was stained with 4′,6-diamidino-2-phenylindole (DAPI; Sigma-Aldrich) dye at a 5 µg/mL concentration for 20 min, washed 3 times with PBS, and then subsequently mounted. Images were collected using a Zeiss LSM880 laser scanning confocal system with the Plan-Apochromat 63x/1 Oil DIC M27 objective lens. The Argon-multiline (458, 488 nm), HeNe (633 nm), and Diode (405 nm) lasers were used. Images were acquired using 8-bit digitization depth, while detectors Offset and Master Gain were kept constant. Also, the same laser power, pinhole, and pixel dwell time were set to maintain consistent conditions for all images being compared.

### 3.6. Cellular Detachment Assay

U-87 MG, A-172, and LN-229 glioblastoma cells as well as A549 and H1299 adenocarcinoma cells were treated with TTFields for 24 h. Then, cells were trypsinized and cellular detachment monitored following trypsin treatment, by calculating the fraction of detached cells over total number of cells in the dish. Duration of trypsinization times was determined by the duration for complete (100%) removal of cells from control dishes and were as follows: U-87 MG, 5 min; A-172, 10 min; LN-229, 15 min; A549, 5 min; and H1299, 4 min. The total number of cells in TTFields treated dishes was determined by a second trypsinization leading to complete removal of the cells from the dish. The overall cell count was calculated by combining cell counts from the two trypsinization rounds.

### 3.7. Image Analysis and Quantification of Cellular Geometry

Cell images were analyzed. In the wound healing assay, all images were initially rotated, such that the frontline is to the right-hand of the cells. Thereafter, cells that approached the frontline and appeared whole in the image were manually segmented, using FIJI ImageJ [33]. A custom Python script using OpenCV [34] was then used to load the segmented cells in continuous succession to analyze geometry and intensities. The following measures were computed for each individual cell: (1) Area; (2) distance between nuclei center and center of mass; (3) overall tubulin intensity; (4) average tubulin intensity; and (5) angle between the major axis and the frontline. Fluorescence intensity data was obtained by defining the region of interest and measuring the average fluorescence intensity. The background intensity, determined by selecting an area lacking microtubules and signals from focal adhesions, was subtracted from these values. The major axis was computed by initially fitting a minimal bounding rectangle to the cell. The direction of the long edge was selected and its angle with respect to the frontline was computed. The frontline was defined as 0 degrees and its perpendicular defined as 90 degrees. Larger values (i.e., between 90 and 180 degrees) were reflected to the range of 0–90 degrees. To evaluate focal adhesions, the cells that appeared whole in the image were manually segmented using FIJI ImageJ. A custom Python script using OpenCV was then used to load the segmented cells in succession and analyze the focal adhesion geometry and intensities. The following measures were computed for each individual cell: (1) Area; (2) total focal adhesions area; and (3) intensity of focal adhesions.

### 3.8. Preparation of Glioblastoma Spheroids

A-172 and U-87MG glioblastoma cells were seeded in 3D CoSeedis agarose-based chip (abc biopply ag, Solothurn, Switzerland) at cell densities of 2.5 × 10³ cells/cm². Two days after cell seeding, media was switched to 2% fetal bovine serum and the cells were maintained for an additional 3 days under low serum conditions. At day 6, spheroids were removed from the agarose-based chip and embedded in Cultrex spheroid invasion extracellular matrix to analyze using a 3D dispersal assay (R&D Systems, Minneapolis, MN, USA). The spheroids were then treated with TTFields for 24 h (A-172) and 48 h (U-87 MG).

### 3.9. Dispersal Area Analysis

Z stack images were taken with the Confocal microscope LSM880 (Carl Zeiss Microscopy GmbH, Jena, Germany). Z stack images were viewed in Imaris software (BitPlane AG, Zürich, Switzerland) and snapshots were taken for further analysis. Dispersal area analysis was performed using Fiji software cand images were analyzed using a Lasso tool. Total area of dispersal and remaining spheroids were measured, and overall dispersal was determined using the following equations:

For A-172 spheres: Dispersal area = [total area 24 h/sphere area 24 h].

For U-87 MG spheres: Dispersal area = [total area 48 h/sphere area 48 h].

### 3.10. Rhoa Activation Assay

To measure RhoA activity (GTP-bound active form), A-172 and LN-229 glioblastoma cells were cultured in serum-free DMEM for 24 h and treated with TTFields for 3, 5, 10, 15, and 30 min. Cells were then lysed and RhoA activity was measured by the RhoA G-LISA activation assay in accordance with the manufacturer’s instructions (Cytoskeleton). Results were expressed as the ratio of OD of treated cells to the OD of untreated cells normalized to total RhoA.

### 3.11. ROCK Activation Assay

To measure ROCK activity, U-87 MG, A-172, and LN-229 glioblastoma cells were treated with TTFields for 24 h. Cells were then lysed and ROCK activity measured, utilizing the ROCK activity assay kit in accordance with the manufacturer’s instructions (Abcam).

### 3.12. Western Blotting

A-172 and LN-229 glioblastoma cells were treated with TTFields for 5 and 10 min, respectively. Cell lysates were collected as was described for the RhoA activation assay. 5% milk was used for the blocking step and washes were done by using TBST buffer (Tris-buffered saline, 0.1% Tween 20). Antibodies were used with the following dilutions in 5% BSA: GEF-H1 rabbit monoclonal 55B6 antibody (dilution 1:1000; 4076, Cell Signaling, Beverly, MA, USA), phospho-GEF-H1 (Ser886) rabbit monoclonal E1L6D antibody (dilution 1:1000; 14143, Cell Signaling), and Glyceraldehyde 3-phosphate dehydrogenase (GAPDH) mouse monoclonal antibody (dilution 1:500; sc-32.233, Santacruz); followed by horseradish peroxidase-conjugated secondary antibody (dilution 1:2000; Goat anti-Rabbit 7074, Cell Signaling and Goat anti-Mouse 7076, Cell Signaling) and a chemiluminescent substrate (WBLUF0100, Sigma-Aldrich). The Fiji ImageJ software was applied to measure the band intensities of Western blots (Figures S1 and S2).

### 3.13. Migration of Immune Cells

All animal studies were approved by the Novocure Internal Animal Care Committee, in accordance with the Technion-Israel Institute of Technology guidelines for the care of laboratory animals. To isolate T cells, spleens from 5–7 week old male C57BL/6 mice were dispersed through a 40 µM nylon cell strainer and red blood cells were lysed with RBC lysis buffer (Biolegend, San Diego, CA, USA). Single-cell suspensions were separated by Mouse Pan T magnetic bead selection (Invitrogen, Carlsbad, CA, USA). The purity of each sample was validated by flow cytometry analysis using Mouse anti-CD3 antibody (clone 17A2, BioLegend). Isolated T cells were cultured in vitro in RPMI 1640 medium supplemented with 10% heat-inactivated FBS (Biological Industries), 2 mM L-glutamine, 1% penicillin/streptomycin (Biological Industries), 2 mM HEPES buffer (Biological Industries), and 50 μM 2-mercaptoethanol (Gibco, Loughborough, UK). For the generation of BMDCs, bone marrow cells were flushed from the femurs and tibias of 5–7 week old C57BL/6 mice under sterile conditions. The tissue was dispersed through a 70 µM nylon cell strainer and red blood cells were lysed with RBC lysis buffer. Bone marrow cells were cultured for 7 days with RPMI 1640 medium supplemented with 10% heat-inactivated FBS, 2 mM L-glutamine, 50mM 2-mercaptoethanol, 10 mM HEPES buffer, 0.4mM sodium pyruvate (Biological Industries), 1% penicillin/streptomycin, and 10 ng/mL recombinant murine GM-CSF (Peprotech, Rocky Hill, NJ, USA). 1 µg/mL Lipopolysaccharides (LPS) (Sigma Aldrich) was added for the last 24 h. The purity of each sample was validated by flow cytometry analysis using Mouse anti-CD11c antibody (clone N418, BioLegend). The migratory potential of CD11c+ dendritic cells and CD3+ T-cells towards 0.5 ug/mL CCL19 (Peprotech) during 3 h of TTFields application (200 kHz) was analyzed using the modified Boyden chamber assay (3 µM pore size; Corning, Corning, NY, USA). For all staining protocols, cell suspensions were incubated in mouse Fc block (anti CD16/CD32 clone 93, BioLegend) prior to staining with antibodies for the cell markers. Viobility 405/452 Fixable Dye (Miltenyi Biotec, Bergisch Gladbach, Germany) was used for the discrimination of dead cells. Data were acquired on MACSQuant Analyzer 10 (Miltenyi Biotec) flow cytometer and analyzed using FlowJo software (Ashland, OR, USA).

### 3.14. Statistical Analysis

Data were analyzed with Graphpad Prism software (Graphpad, San Diego, CA, USA), and *p* values of < 0.05 were considered to be statistically significant and indicated as * *p*  <  0.05, ** *p*  <  0.01, and *** *p*  <  0.001.

## 4. Conclusions

**Figure 6:**
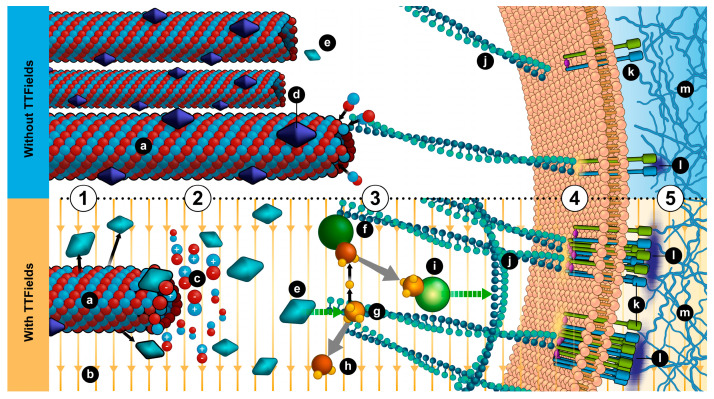
A model illustrating the mechanism by which TTFields modulates cancer cell motility**. (1)** Microtubules are required to specify the direction of cell movement. GEF-H1 catalytic activity is downregulated through microtubule binding. **(2)** TTFields exert directional forces on polar tubulins leading to their alignment in the direction of the field. This in turn, leads to the reorganization of the microtubule network resulting in changes in the number of microtubules, interference with the directionality of cellular migration, and initiation of the GEF-H1/RhoA/ROCK signaling pathway **(3)** to control actin dynamics **(4)** and formation of focal adhesions **(5),** which disrupt cell polarity and migration directionality. **a**, microtubule; **b**, TTFields; **c**, tubulin aligned with field; **d**, GEF-H1; **e**, active GEF-H1; **f**, Rho-A GDP; ***g***, GTP; ***h***, GDP; **i**, Rho-A GTP; **j**, actin fiber; **k**, integrin; **l**, focal adhesion; **m**, extracellular matrix.

In this study, we demonstrated that similar to the effects of TTFields on mitotic spindle microtubules, TTFields also reduced the quantity of microtubules and dictated the directionality of cancer migration. These changes in microtubule organization also led to increased activation of GEF-H1, actuating the RhoA/ROCK signaling cascade and further inducing the formation of a dense meshwork of peripheral actin filaments and an increase in size and number of focal adhesions (Figure 6).

In addition, our data suggests that in cells exposed to TTFields, the microtubules tend to align with the field, further inhibiting the ability of cells to migrate freely. Electro-orientation of microtubules in solution in response to alternating electric fields has been reported [35]. However, the field intensities at which this occurs are at least three orders of magnitude higher than the field intensities of TTFields. Alignment of microtubules in low intensity DC electric fields has been observed in kinesin-propelled microtubule assays [36]. The combination of these observations provides a basis on which a theory explaining the alignment of cellular microtubules in response to TTFields can be constructed, and should continue to be investigated. In this study, we also demonstrated that TTFields application does not impair leukocyte migration; however, the integrity and function of the microtubule and actin cytoskeleton are critical for the function of many other healthy tissues. Although we have not tested the effect of TTFields on cytoskeletal architecture of non-cancerous cells, data from clinical trials and extensive safety studies in healthy animals have shown that TTFields are not associated with significant systemic toxicities [7,37]. Collectively, our study provides a possible unifying molecular mechanism connecting the already established TTFields effect on microtubule disruption to changes in actin organization and cellular polarity that is required for effective cancer cell motility.

## Supplementary Materials

The following are available online at https://www.mdpi.com/2072-6694/12/10/3016/s1, Figure S1: LN229 cells - GEFH1 whole Western blot, Figure S2: A172 cells - GEFH1 whole Western blot, Video S1: Time lapse of wound healing in TTFields treated U-87 MG cells, Video S2: Time lapse of wound healing in TTFields treated A-172 cells, Video S3: Time lapse of wound healing in A-172 cells treated with either bi or uni-directional TTFields.
