# Supplementary material for: Tumor Treating Fields (TTFields) Hinder Cancer Cell Motility through Regulation of Microtubule and Actin Dynamics
Source: Cancers (Basel). 2020 Oct 17;12(10):3016. doi: 10.3390/cancers12103016 (PMC7603026; doi:10.3390/cancers12103016)
Supplement: Supplementary file 1 [file cancers-12-03016-s001.zip › cancers-956966 suppl final.docx]

**Supplementary Materials:**

Tumor Treating Fields (TTFields) Hinder Cancer Cell Motility through Regulation of Microtubule and Acting Dynamics


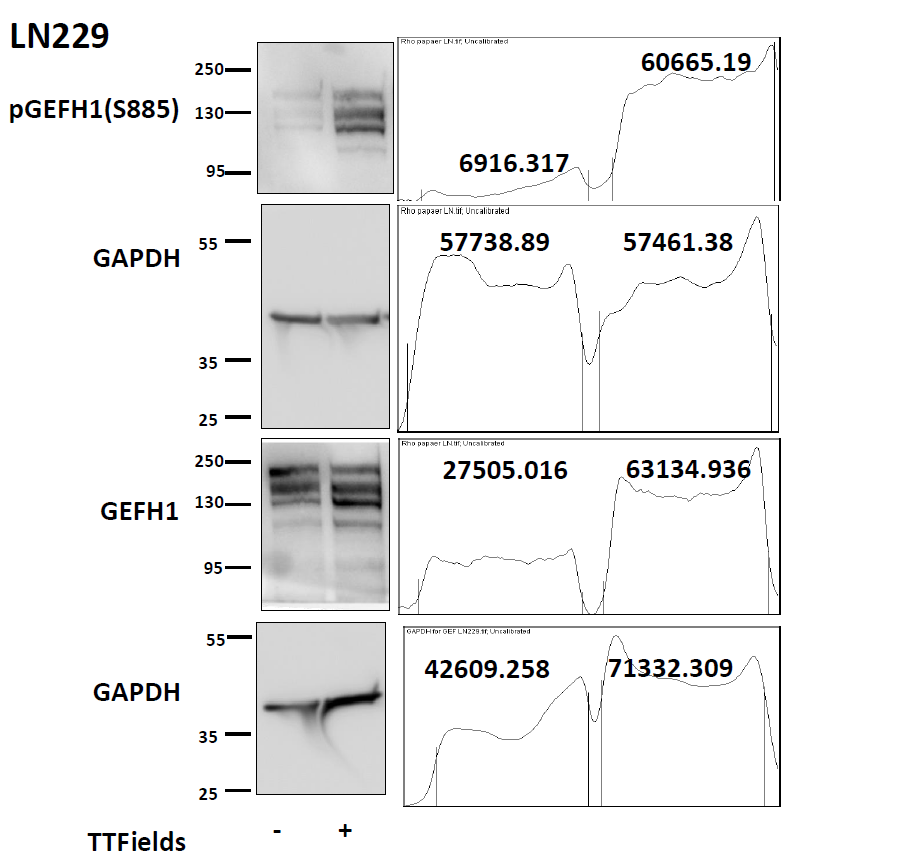


**Figure S1**. LN229 cells - GEFH1 whole Western blot


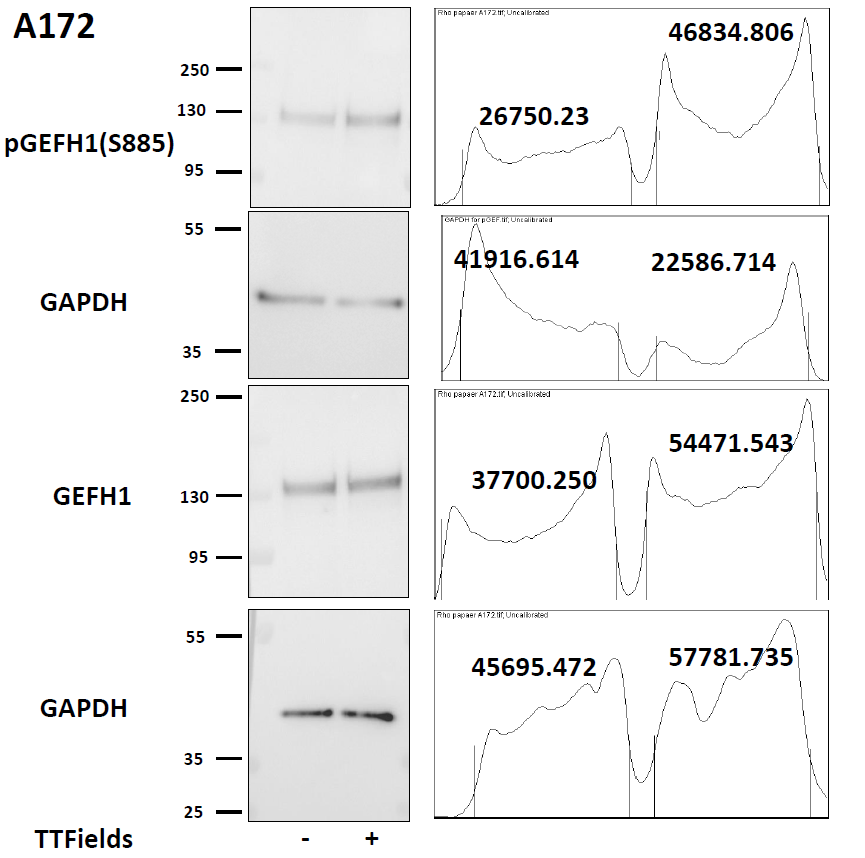


**Figure S2.** A172 cells - GEFH1 whole Western blot

Supplementary Video S1- Time lapse of wound healing in TTFields treated U-87 MG cells.

Supplementary Video S2- Time lapse of wound healing in TTFields treated A-172 cells.

Supplementary Video S3- Time lapse of wound healing in A-172 cells treated with either bi or uni-directional TTFields.
